# Near-Infrared Spectroscopy Detects Lipid-Rich Plaque Preceding ST-Segment Elevation Myocardial Infarction

**DOI:** 10.1016/j.jscai.2025.103990

**Published:** 2025-10-07

**Authors:** Shazil Mahmood, Samia Mazumder, Ryan D. Madder

**Affiliations:** Department of Cardiovascular Medicine, William Beaumont University Hospital, Corewell Health East, Royal Oak, Michigan

**Keywords:** case report, intravascular ultrasound, lipid core burden index, near-infrared spectroscopy

## Case report

**Figure 1:**
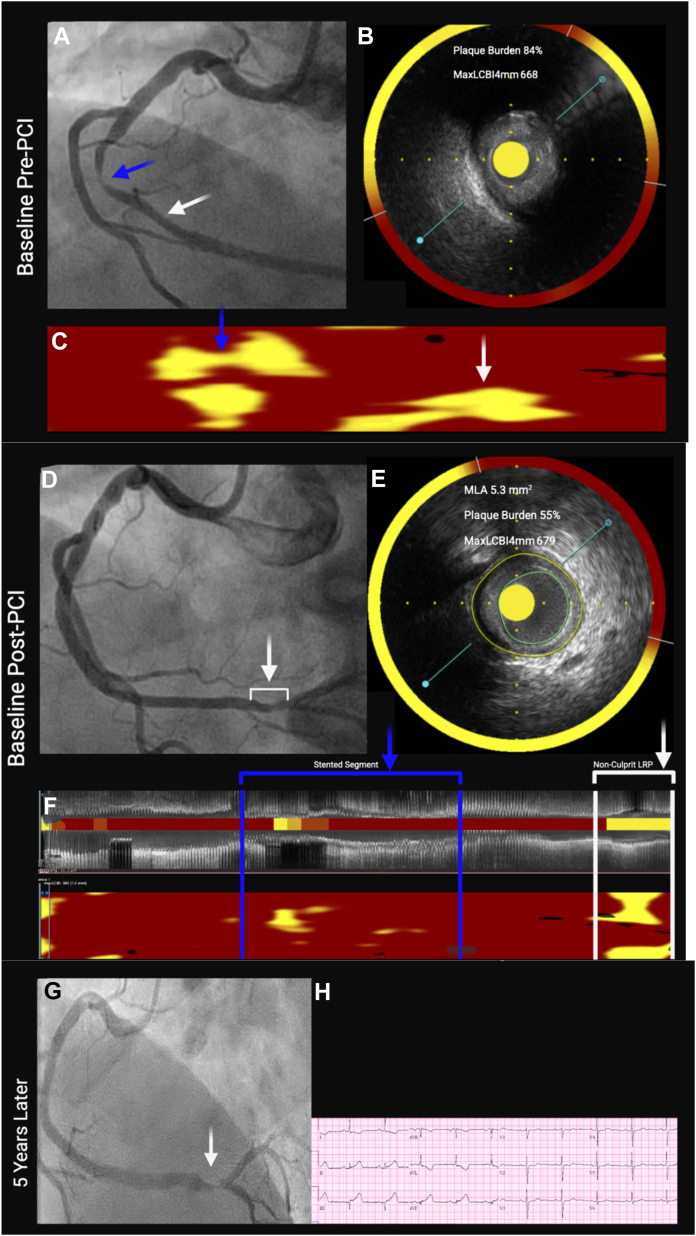
**Near-infrared spectroscopy (NIRS) lipid-rich plaque (LRP) at baseline followed byST-segment elevation myocardial infarction (STEMI) 5 years later.** (**A**) Baseline angiogram—blue arrow shows culprit lesion and white arrow shows mild angiographic stenosis. (**B**) Cross-sectional NIRS-intravenous ultrasound (IVUS) image of culprit lesion showing large plaque burden (PB) and large LRP. (**C**) NIRS chemogram showing culprit lesion LRP (blue arrow) and lipid corresponding to site of mild angiographic stenosis (white arrow). (**D**) Final angiogram at conclusion of baseline percutaneous coronary intervention (PCI). White bracket and arrow show nonculprit lesion with moderate angiographic stenosis. (**E**) Cross-sectional NIRS-IVUS image of nonculprit lesion in distal right coronary artery (RCA) showing adequate minimum luminal area (MLA) and moderate PB by IVUS and a high maxLCBI_4mm_ by NIRS. (**F**) NIRS chemogram showing stenting segment (blue brackets) and large LRP at nonculprit site in distal RCA (white brackets). (**G**) Angiogram at time of STEMI presentation 5 years later showing a culprit lesion in the distal RCA. (**H**) Electrocardiogram showing inferior STEMI.

A 57-year-old woman with unstable angina underwent invasive coronary angiography demonstrating a culprit lesion in the midsegment of the right coronary artery (RCA) (Figure 1A). Combined near-infrared spectroscopy (NIRS) and intravascular ultrasound (IVUS) imaging of the culprit lesion before stent placement demonstrated a plaque burden (PB) of 84% by IVUS and a large lipid-rich plaque (LRP) by NIRS characterized by a maximum lipid core burden index in 4 mm (maxLCBI_4mm_) of 668 (Figure 1B). NIRS also demonstrated LRP extending beyond the distal angiographic margin of the culprit lesion at the site of mild angiographic stenosis (Figure 1C). The patient underwent successful percutaneous coronary intervention (PCI) using a stent of sufficient length to cover both the culprit lesion and the adjacent LRP. Post-PCI NIRS-IVUS demonstrated an optimal stent result. Final angiography showed a moderate severity nonculprit lesion in the distal RCA just proximal to the bifurcation (Figure 1D). Review of the post-PCI NIRS-IVUS images revealed this nonculprit lesion had a minimum luminal area (MLA) of 5.3 mm^2^, a PB of 55%, and a large lipid burden with a maxLCBI_4mm_ of 679 (Figure 1E, F). Owing to an MLA of 5.3 mm^2^ by IVUS and only moderate stenosis severity by angiography, PCI was not performed on this nonculprit lesion. The patient was discharged home on dual antiplatelet therapy and a high-intensity statin.

Approximately 5 years later, the patient presented to the emergency room with acute chest pain and was found to have an inferior ST-segment elevation myocardial infarction (STEMI). She was taken emergently to the catheterization laboratory and found to have a de novo culprit lesion in the distal RCA just proximal to the bifurcation at the site of the large LRP detected by NIRS 5 years earlier (Figure 1G, H). The patient underwent successful primary PCI of the culprit lesion in the distal RCA.

## Discussion

This case emphasizes multiple known facts about NIRS findings at culprit and nonculprit lesions and highlights several knowledge gaps that exist with respect to NIRS-detected LRP. Among the known facts, multiple previous studies among patients with acute coronary syndromes (ACS) have shown NIRS frequently identifies large LRP having a maxLCBI_4mm_ >400 at culprit lesion sites. Consistent with these studies, the culprit lesion triggering the index ACS in the present case had a NIRS maxLCBI_4mm_ of 668. In addition to its consistency with previous NIRS studies, this finding is also congruent with histopathologic studies demonstrating the rupture of large LRP to be responsible for the majority of ACS events. In the present case, NIRS also detected LRP extending beyond the angiographic culprit lesion margins, a finding previously reported to be commonplace. This observation emphasizes the importance of using intracoronary imaging to identify the optimal landing zones for stent deployment, as landing a stent edge within an LRP has been associated with an increased risk of subsequent stent-related events.^1^ Based on this premise, a longer stent was used during the baseline PCI to cover both the culprit lesion and adjacent LRP.

Perhaps, the most interesting aspect of this case relates to the imaging findings of the nonculprit lesion in the distal RCA at the conclusion of the baseline procedure. Neither the moderate stenosis severity by angiography at the nonculprit site nor the IVUS MLA of 5.3 mm^2^, which is well above IVUS MLA thresholds known to be associated with hemodynamic significance, would represent indications for PCI at the time the baseline case was performed. Furthermore, the PB of 55% is not in excess of the 70% PB associated with vulnerability. In contrast to the lower-risk IVUS findings, the NIRS finding of a maxLCBI_4mm_ of 679 at the nonculprit site is indicative of both increased patient-level and lesion-level risk. As shown in the Lipid-Rich Plaque study, plaques having a maxLCBI_4mm_ >400 carry a 4-fold increase of site-specific coronary events.^2^ Similarly, in the PROSPECT II study, plaques having a maxLCBI_4mm_ >325 were associated with a 7-fold increased risk of site-specific major adverse cardiovascular events.^3^

While consistent with the findings of the Lipid-Rich Plaque and PROSPECT II studies, this case also highlights several uncertainties regarding NIRS-detected LRP. First, the follow-up periods in the Lipid-Rich Plaque and PROSPECT II studies were 2 and 3.7 years, respectively. However, in the present case, a STEMI culprit lesion emerged at the site of a high-risk NIRS plaque detected 5 years earlier, a timeframe outside the follow-up periods of the Lipid-Rich Plaque and PROSPECT II studies. This observation raises important questions regarding the duration of increased risk posed by large LRP detected by NIRS. Second, there is considerable debate regarding the merit of preemptive PCI of NIRS-detected high-risk plaques, such as the large nonculprit LRP found in the present case. It is notable in the PREVENT study that preventative PCI of non–flow-limiting vulnerable plaques, including those identified by NIRS, was associated with a reduction in subsequent clinical events.^4^ However, the number of PCIs needed to prevent 1 clinical event was high in PREVENT study, and the cost effectiveness of this approach is unknown. Finally, it remains uncertain whether starting more aggressive lipid-lowering therapies should be implemented when high-risk nonculprit plaques are found by NIRS. The PACMAN-AMI trial demonstrated early initiation of alirocumab, in combination with high-intensity statin therapy, significantly enhanced coronary plaque regression over a 52-week period.^5^ Whether this approach will result in lower clinical event rates requires further study.

## Focus points

• Culprit lesions in ACS commonly have a NIRS maxLCBI_4mm_ >400.
• NIRS-detected LRP frequently extends beyond the angiographic culprit lesion margins.
• Landing a stent edge within an LRP has been associated with an increased risk of subsequent stent-related events.
• Nonculprit plaques with an elevated NIRS maxLCBI_4mm_ carry an increased risk of triggering future site-specific coronary events.
• Preventative PCI of non–flow-limiting vulnerable plaques, including those identified by NIRS, has been shown to reduce subsequent clinical events; however, the number of PCIs needed to prevent 1 clinical event is high.
